# Supplementary figures and images for: Prognostic value of high-sensitivity cardiac troponin I in heart failure patients with mid-range and reduced ejection fraction
Source: PLoS One. 2021 Jul 30;16(7):e0255271. doi: 10.1371/journal.pone.0255271 (PMC8323897; doi:10.1371/journal.pone.0255271)

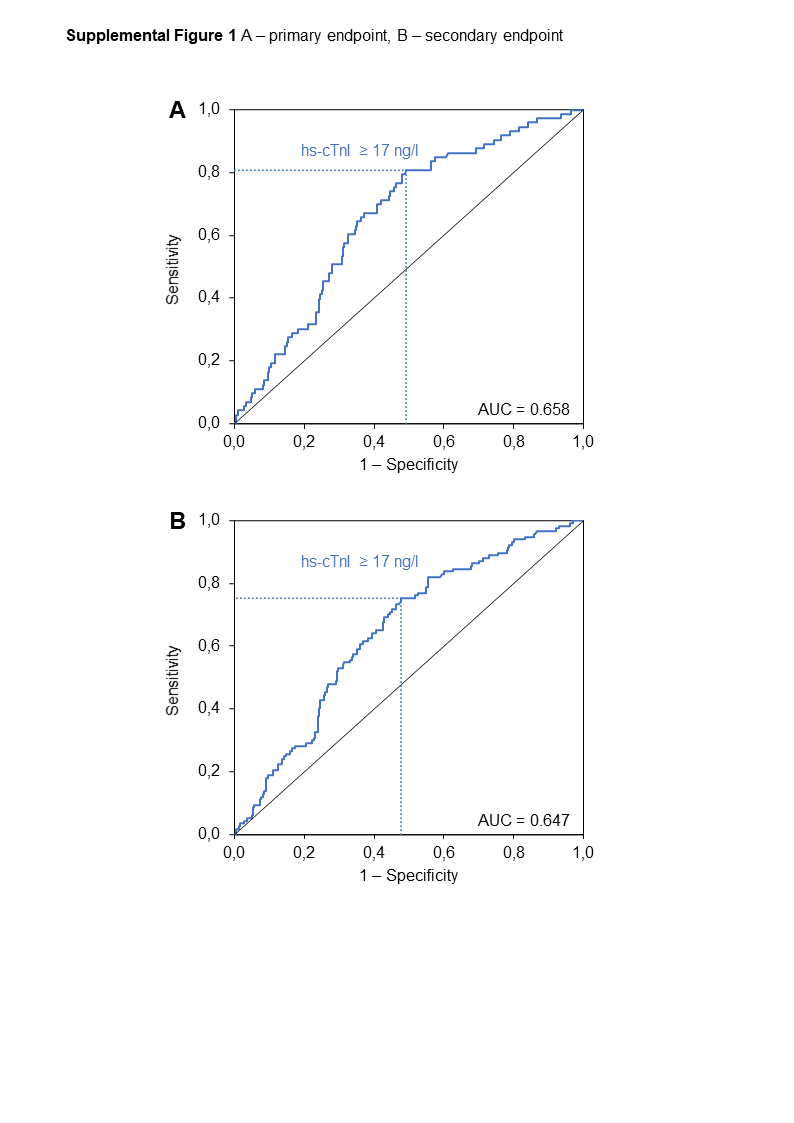

Supplement: S1 Fig — (TIF) [file pone.0255271.s001.tif]

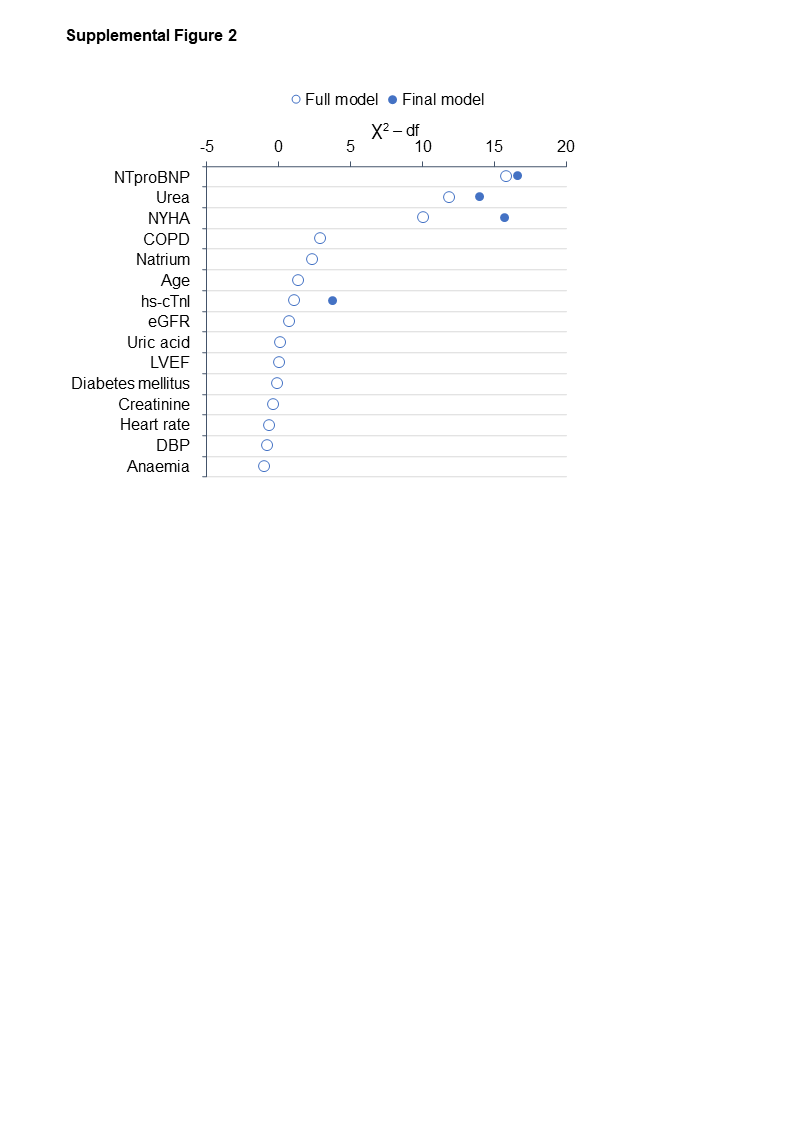

Supplement: S2 Fig — (TIF) [file pone.0255271.s002.tif]

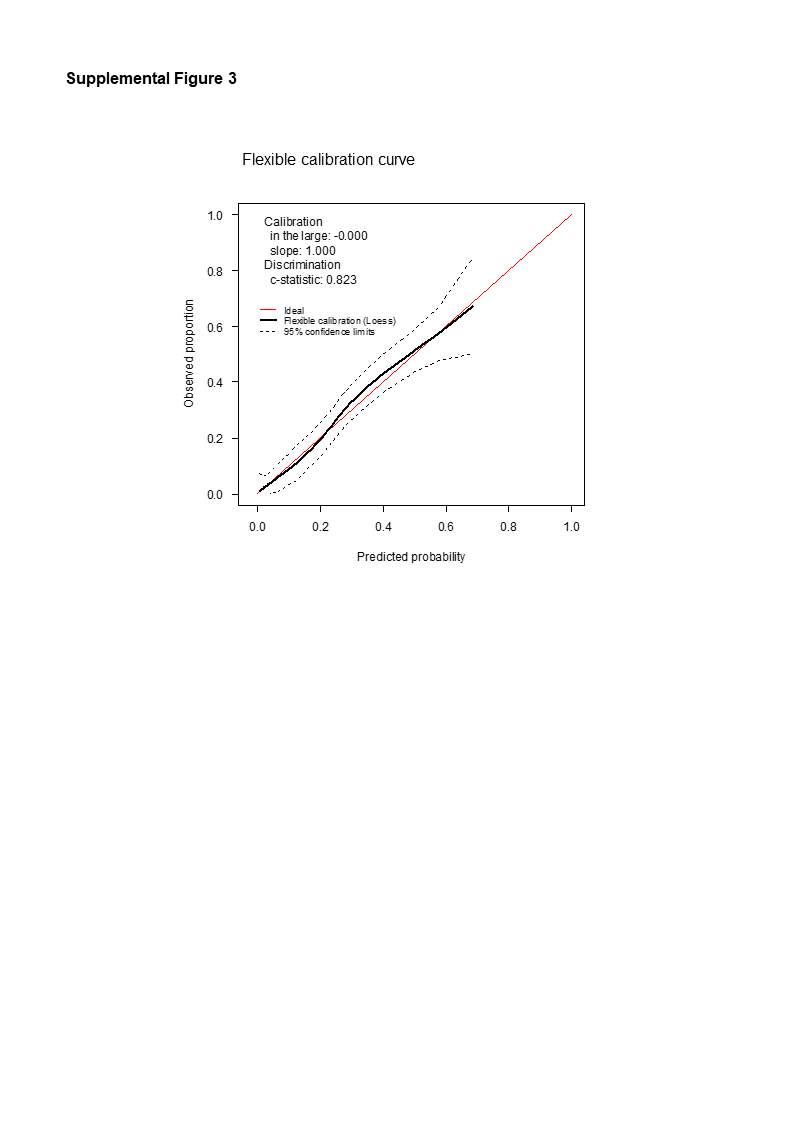

Supplement: S3 Fig — (TIF) [file pone.0255271.s003.tif]
