# Supplementary material for: Prognostic value of high-sensitivity cardiac troponin I in heart failure patients with mid-range and reduced ejection fraction
Source: PLoS One. 2021 Jul 30;16(7):e0255271. doi: 10.1371/journal.pone.0255271 (PMC8323897; doi:10.1371/journal.pone.0255271)
Supplement: S1 Table — (DOCX) [file pone.0255271.s004.docx]

**S1 Table:** Summary of event occurrence in the first two years of follow-up

|  | **n (%)** |
| --- | --- |
| **Occurrence of event** |  |
| Death | 57 (11.0%) |
| LVAD | 8 (1.5%) |
| HTX | 13 (2.5%) |
| Hospitalization for HF | 74 (14.2%) |
| **Combination of events** |  |
| Hospitalization for HF only | 44 (8.5%) |
| Death only | 37 (7.1%) |
| Hospitalization for HF + death | 18 (3.5%) |
| Hospitalization for HF + HTX | 6 (1.2%) |
| HTX only | 4 (0.8%) |
| Hospitalization for HF + LVAD + HTX | 3 (0.6%) |
| LVAD only | 2 (0.4%) |
| Hospitalization for HF + LVAD + death | 2 (0.4%) |
| Hospitalization for HF + LVAD | 1 (0.2%) |
| None | 403 (77.5%) |

LVAD , left ventricular assist device; HF – heart failure; HTX – heart transplantation
