# Supplementary material for: Prognostic value of high-sensitivity cardiac troponin I in heart failure patients with mid-range and reduced ejection fraction
Source: PLoS One. 2021 Jul 30;16(7):e0255271. doi: 10.1371/journal.pone.0255271 (PMC8323897; doi:10.1371/journal.pone.0255271)
Supplement: S2 Table — (DOCX) [file pone.0255271.s005.docx]

**S2 Table:** Basic characteristics of the patients according to occurrence of secondary endpoint (i.e., the two-year prognosis in terms of all-cause mortality, heart transplantation, left ventricular assist device [LVAD] implantation, hospitalization for HF)

| **Parameter** | **Total (N = 520)** | **Without endpoint (n = 403)** | **With endpoint (n = 117)** | **P-value** |
| --- | --- | --- | --- | --- |
| **Basic characteristics** |  |  |  |  |
| Sex – male | 419 (80.6%) | 324 (80.4%) | 95 (81.2%) | NS |
| Age | 65 ± 12 | 65 ± 12 | 66 ± 12 | NS |
| BMI | 29 ± 5 | 29 ± 5 | 29 ± 5 | NS |
| SBP [mmHg] | 127 ± 15 | 129 ± 15 | 123 ± 15 | **0.007** |
| DBP [mmHg] | 80 ± 10 | 81 ± 11 | 77 ± 10 | NS |
| Heart rate [min^-1^] | 73 ± 13 | 72 ± 12 | 76 ± 14 | NS |
| LVEF [%] | 32 ± 9 | 33 ± 9 | 28 ± 8 | **< 0.001** |
| Ischaemic aetiology of HF | 283 (54.4%) | 223 (55.3%) | 60 (51.3%) | NS |
| Hypertension | 344 (66.2%) | 257 (63.8%) | 87 (74.4%) | NS |
| Atrial fibrillation | 173 (33.3%) | 126 (31.3%) | 47 (40.2%) | NS |
| Diabetes mellitus | 205 (39.4%) | 149 (37.0%) | 56 (47.9%) | NS |
| COPD | 80 (15.4%) | 55 (13.6%) | 25 (21.4%) | NS |
| Lower extremity peripheral artery disease | 49 (9.4%) | 34 (8.4%) | 15 (12.8%) | NS |
| Smoking |  |  |  | NS |
| Non-smoker | 298 (57.3%) | 229 (56.8%) | 69 (59.0%) |  |
| Smoker | 55 (10.6%) | 47 (11.7%) | 8 (6.8%) |  |
| Ex-smoker | 167 (32.1%) | 127 (31.5%) | 40 (34.2%) |  |
| NYHA classification |  |  |  | **< 0.001** |
| 1 | 75 (14.4%) | 69 (17.1%) | 6 (5.1%) |  |
| 2 | 349 (67.1%) | 276 (68.5%) | 73 (62.4%) |  |
| 3–4 | 96 (18.5%) | 58 (14.4%) | 38 (32.5%) |  |
| **Laboratory results** |  |  |  |  |
| hs-cTnI [ng/l] | 19 (4; 339) | 16 (4; 311) | 31 (6; 374) | **< 0.001** |
| NT-proBNP [ng/l] | 690 (44; 6,038) | 444 (36; 3 798) | 1 865 (224; 11 912) | **< 0.001** |
| Haemoglobin [g/l] | 144 (114; 167) | 146 (116; 169) | 137 (106; 160) | **< 0.001** |
| Natrium [mmol/l] | 141 (135; 146) | 141 (135; 146) | 141 (133; 146) | NS |
| Urea [mmol/l] | 6 (4; 15) | 6 (4; 12) | 8 (4; 21) | **< 0.001** |
| Uric acid [µmol/l] | 397 (234; 592) | 388 (236; 568) | 420 (221; 633) | NS |
| Creatinine [μmol/l] | 95 (66; 176) | 94 (67; 167) | 101 (63; 222) | NS |
| eGFR [ml/min/1.73 m^2^] | 70 (29; 103) | 71 (33; 103) | 65 (21; 103) | NS |
| **Medication** |  |  |  |  |
| ACEI/ARB | 464 (89.2%) | 362 (89.8%) | 102 (87.2%) | NS |
| Beta-blockers | 485 (93.3%) | 378 (93.8%) | 107 (91.5%) | NS |
| Furosemide ≥ 40 mg/day | 296 (56.9%) | 208 (51.6%) | 88 (75.2%) | **< 0.001** |
| Spironolactone/eplerenone | 334 (64.2%) | 249 (61.8%) | 85 (72.6%) | NS |

The categorical variables are characterised by absolute and relative frequencies. The continuous basic characteristics are described as the mean ± SD, and laboratory results are described as the median (5th–95th percentile).
BMI, body mass index; COPD, chronic obstructive pulmonary disease; DBP, diastolic blood pressure; HF, heart failure; LVEF, left ventricular ejection fraction; SBP, systolic blood pressure; eGFR, estimated glomerular filtration rate (using the CKD-EPI equation).
The p-value of the Fisher’s exact test for categorical variables and the p-value of the Mann-Whitney U test are shown with the Bonferroni correction applied.
