# Supplementary material for: Prognostic value of high-sensitivity cardiac troponin I in heart failure patients with mid-range and reduced ejection fraction
Source: PLoS One. 2021 Jul 30;16(7):e0255271. doi: 10.1371/journal.pone.0255271 (PMC8323897; doi:10.1371/journal.pone.0255271)
Supplement: S3 Table — (DOCX) [file pone.0255271.s006.docx]

**S3 Table:** Comparison of hs-cTnI between patient subgroups

| **Parameter** |  | **hs-cTnI [ng/l]** | | **P-value** |
| --- | --- | --- | --- | --- |
|  |  | **n** | **Median (5th–95th percentile)** |  |
| Sex | Men | 419 | 18.8 (4.6; 310.8) | NS |
|  | Women | 101 | 18.7 (2.8; 406.5) |  |
| Age | < 65 years | 238 | 14.9 (2.9; 265.4) | **< 0.001** |
|  | ≥ 65 years | 282 | 22.0 (5.8; 362.0) |  |
| BMI | < 30 | 316 | 18.5 (4.0; 352.6) | NS |
|  | ≥ 30 | 200 | 18.9 (4.6; 270.1) |  |
| LVEF | < 35 % | 300 | 24,1 (5,0; 361,2) | **< 0.001** |
|  | ≥ 35 % | 220 | 12,9 (3,2; 250,9) |  |
| Ischaemic aetiology of HF | No | 237 | 17.3 (3.9; 310.8) | NS |
|  | Yes | 283 | 19.7 (4.7; 361.9) |  |
| Hypertension | No | 176 | 15.7 (3.0; 361.9) | NS |
|  | Yes | 344 | 19.4 (5.0; 280.2) |  |
| Atrial fibrillation | No | 347 | 18.1 (3.7; 272.2) | NS |
|  | Yes | 173 | 19.9 (5.8; 360.6) |  |
| Diabetes mellitus | No | 315 | 17.0 (3.9; 332.1) | NS |
|  | Yes | 205 | 20.4 (5.1; 345.6) |  |
| COPD | No | 440 | 18.8 (4.1; 360.5) | NS |
|  | Yes | 80 | 17.8 (5.0; 113.2) |  |
| Lower extremity | No | 471 | 18.2 (4.0; 352.6) | NS |
| peripheral artery disease | Yes | 49 | 26.0 (6.8; 171.1) |  |
| eGFR | < 60 ml/min/1.73m^2^ | 179 | 27.9 (6.2; 507.4) | **< 0.001** |
|  | ≥ 60 ml/min/1.73m^2^ | 341 | 15.8 (3.5; 261.7) |  |

The p-value of Mann-Whitney U test is shown with Bonferroni correction applied. BMI, body mass index; COPD, chronic obstructive pulmonary disease; HF, heart failure; LVEF, left ventricular ejection fraction; eGFR, estimated glomerular filtration rate (using the CKD-EPI equation).
