# Supplementary material for: Prognostic value of high-sensitivity cardiac troponin I in heart failure patients with mid-range and reduced ejection fraction
Source: PLoS One. 2021 Jul 30;16(7):e0255271. doi: 10.1371/journal.pone.0255271 (PMC8323897; doi:10.1371/journal.pone.0255271)
Supplement: S4 Table — (DOCX) [file pone.0255271.s007.docx]

**S4 Table:** Correlation between hs-cTnI and other parameters

|  | **Spearman's correlation coefficient** | **P-value** |
| --- | --- | --- |
| Age | 0.186 | **< 0.001** |
| BMI | -0.047 | NS |
| SBP | -0.034 | NS |
| DBP | -0.071 | NS |
| Heart rate | 0.198 | **< 0.001** |
| LVEF | -0.208 | **< 0.001** |
| NT-proBNP | 0.439 | **< 0.001** |
| Haemoglobin | -0.113 | NS |
| Urea | 0.241 | **< 0.001** |
| Creatinine | 0.245 | **< 0.001** |
| eGFR | -0.240 | **< 0.001** |

The statistical signification of correlation coefficient is shown with Bonferroni correction applied. BMI, body mass index; DBP, diastolic blood pressure; LVEF, left ventricular ejection fraction; SBP, systolic blood pressure; eGFR, estimated glomerular filtration rate (using the CKD-EPI equation).
