# Supplementary material for: Prognostic value of high-sensitivity cardiac troponin I in heart failure patients with mid-range and reduced ejection fraction
Source: PLoS One. 2021 Jul 30;16(7):e0255271. doi: 10.1371/journal.pone.0255271 (PMC8323897; doi:10.1371/journal.pone.0255271)
Supplement: S5 Table — (DOCX) [file pone.0255271.s008.docx]

**S5 Table:** Accuracy of the multivariable prediction model

| **Predictor** | **AUC (95% CI)** | **P** | **Sens.** | **Spec.** | **PPV** | **NPV** | **OA** |
| --- | --- | --- | --- | --- | --- | --- | --- |
| Multivariaable model | 0.823 (0.773; 0.872) | **< 0.001** | 77.5% | 74.4% | 32.7% | 95.3% | 74.8% |

AUC = area under curve, Sens. = sensitivity, Spec. = specificity, PPV = positive predictive value, NPV = negative predictive value, OA = overall accuracy.
