# Supplementary material for: Prognostic value of high-sensitivity cardiac troponin I in heart failure patients with mid-range and reduced ejection fraction
Source: PLoS One. 2021 Jul 30;16(7):e0255271. doi: 10.1371/journal.pone.0255271 (PMC8323897; doi:10.1371/journal.pone.0255271)
Supplement: S6 Table — (DOCX) [file pone.0255271.s009.docx]

**S6 Table:** The multivariable logistic regression model using a backward stepwise algorithm for the selection of independent predictors of the primary endpoint (i.e., the two-year prognosis in terms of all-cause mortality, heart transplantation, left ventricular assist device [LVAD] implantation, hospitalization for HF)

| **Predictor** |  | **OR (95% CI)** | **P** |
| --- | --- | --- | --- |
| NYHA | > 2 (ref. ≤ 2) | 2.64 (1.54; 4.52) | **< 0.001** |
| NT-proBNP [ng/l] | 1-category increase* | 1.84 (1.55; 2.19) | **< 0.001** |
| Urea [mmol/l] | 1-category increase** | 1.78 (1.28; 2.49) | **< 0.001** |

*Categories of NT-proBNP: < 100 / 100–249 / 250–499 / 500–999 / 1,000–1,999 / ≥ 2,000
**Categories of urea: < 6 / 6–9.9 / ≥ 10

AUC = 0.802 (95% CI: 0.761–0.847)
