# Supplementary material for: Prognostic value of high-sensitivity cardiac troponin I in heart failure patients with mid-range and reduced ejection fraction
Source: PLoS One. 2021 Jul 30;16(7):e0255271. doi: 10.1371/journal.pone.0255271 (PMC8323897; doi:10.1371/journal.pone.0255271)
Supplement: S7 Table — (DOCX) [file pone.0255271.s010.docx]

**S7 Table:** Basic characteristics of patients with HFrEF/HFmrEF

| **Parameter** | **HRrEF (< 40%) (n = 400)** | **HRmrEF (40–49%) (n = 120)** | **P-value** |
| --- | --- | --- | --- |
| **Basic characteristics** |  |  |  |
| Sex – male | 335 (83.8%) | 84 (70.0%) | **0.041** |
| Age | 64 ± 12 | 68 ± 11 | **0.012** |
| BMI | 29 ± 5 | 29 ± 4 | NS |
| SBP [mmHg] | 126 ± 15 | 132 ± 17 | **0.015** |
| DBP [mmHg] | 80 ± 11 | 81 ± 9 | NS |
| Heart rate [min^-1^] | 74 ± 12 | 71 ± 13 | **0.043** |
| Ischaemic aetiology of HF | 197 (49.3%) | 86 (71.7%) | **< 0.001** |
| Hypertension | 264 (66.0%) | 80 (66.7%) | NS |
| Atrial fibrillation | 134 (33.5%) | 39 (32.5%) | NS |
| Diabetes mellitus | 167 (41.8%) | 38 (31.7%) | NS |
| COPD | 61 (15.3%) | 19 (15.8%) | NS |
| Lower extremity peripheral artery disease | 38 (9.5%) | 11 (9.2%) | NS |
| Smoking |  |  | NS |
| Non-smoker | 223 (55.8%) | 75 (62.5%) |  |
| Smoker | 42 (10.5%) | 13 (10.8%) |  |
| Ex-smoker | 135 (33.8%) | 32 (26.7%) |  |
| NYHA classification |  |  | **< 0.001** |
| 1 | 41 (10.3%) | 34 (28.3%) |  |
| 2 | 270 (67.5%) | 79 (65.8%) |  |
| 3–4 | 89 (22.3%) | 7 (5.8%) |  |
| **Laboratory results** |  |  |  |
| hs-cTnI [ng/l] | 20 (5; 360) | 12 (3; 260) | **0.013** |
| NT-proBNP [ng/l] | 863 (51; 6 258) | 339 (36; 2 910) | **< 0.001** |
| Haemoglobin [g/l] | 144 (114; 168) | 146 (114; 165) | NS |
| Natrium [mmol/l] | 141 (135; 146) | 142 (135; 145) | NS |
| Urea [mmol/l] | 6 (4; 16) | 6 (4; 12) | NS |
| Uric acid [µmol/l] | 406 (234; 604) | 374 (232; 553) | **0.042** |
| Creatinine [μmol/l] | 97 (68; 180) | 91 (63; 169) | NS |
| eGFR [ml/min/1.73 m^2^] | 69 (29; 103) | 71 (30; 100) | NS |
| **Medication** |  |  |  |
| ACEI/ARB | 351 (87.8%) | 113 (94.2%) | NS |
| Beta-blockers | 380 (95.0%) | 105 (87.5%) | NS |
| Furosemide ≥ 40 mg/day | 261 (65.3%) | 35 (29.2%) | **< 0.001** |
| Spironolactone/eplerenone | 291 (72.8%) | 43 (35.8%) | **< 0.001** |
| **Endpoint** |  |  |  |
| Yes (death, LVAD, HTX within 2 years) | 63 (15.8%) | 10 (8.3%) | NS |

The categorical variables are characterised by absolute and relative frequencies. The continuous basic characteristics are described as the mean ± SD, and laboratory results are described as the median (5th–95th percentile).
BMI, body mass index; COPD, chronic obstructive pulmonary disease; DBP, diastolic blood pressure; HF, heart failure; SBP, systolic blood pressure.
The p-value of the Fisher’s exact test for categorical variables and the p-value of the Mann-Whitney U test are shown with the Bonferroni correction applied.
