# Supplementary material for: Prognostic value of high-sensitivity cardiac troponin I in heart failure patients with mid-range and reduced ejection fraction
Source: PLoS One. 2021 Jul 30;16(7):e0255271. doi: 10.1371/journal.pone.0255271 (PMC8323897; doi:10.1371/journal.pone.0255271)
Supplement: Attachment — Submitted filename: Answers_to_Comments_02 (1).docx [file pone.0255271.s011.docx]

**Reviewer #1**: In this study, the authors examined the association of hs-cTnI and prognosis in 520 patients hospitalized with stable chronic heart failure. The main findings were that hs-cTnI correlated with age, LVEF, and renal function, and they correlate with the NT-proBNP levels in study patients. The value of hs-cTnI also identify the patients that are at a higher risk of a combined endpoint. Those data are felt to be interesting. However, this paper might be improved if the authors could reconsider the following points.

Major Comments
#1 The authors should show in the definition of chronic heart failure. Because there is no mention of who made the diagnosis or the echocardiography results made the diagnosis. Were these patient's enrolled the A&E department, cardiology clinic, medical team etc? What is the accuracy of diagnosis? This is the most important potential bias in the study.

We changed and improved the section Study population, please see pp 4-5.

#2 The authors how to think about the timing of the measurement of hs-cTnI for their conclusion. Anytime is OK?

Thank you for this very important point. Our results respond to hs-cTnI measurements in chronic heart failure patients in a stable phase in outpatient clinics, please see section Limitations, p. 16.

#3 The authors should show the figure of ROC curves, because they determine the cut off level of hs-cTnI from the ROC curve. The readers probably confirm the difference of cut off level of hs-cTnI affect the prognosis. The cutoff level of hs-cTnI >17ng is not a strong predictor because the median hs-cTnI level is 17 ng in the group of "Without endpoint"

We added Supplemental figure 1 – ROC curve. We agree that hs-cTnI alone is not a strong predictor of prognosis. This issue is discussed in the Discussion section, for example on p. 13: *Third, although the predictive value of the hs-cTnI level by itself, as expressed by the C-statistics, is relatively low, adding the hs-cTnI level to the NT-proBNP level and clinical parameters significantly improves the identification of patients that are at a higher risk of a combined endpoint (death, heart transplantation or LVAD implantation).* From both analytical and clinical points of view, the value of hs-cTnI under 17 ng/l also helps to identify low-risk patients.

Other comments
#4 The reviewer cannot understand why it is divided the patients into HFrEF and HFmrEF in this study. Is the prognosis different between HFrEF and HFmrEF? Has the prognostic factor or troponin I cutoff changed?

The study was designed for heart failure patients with HFrEF and HFmrEF. In the patient description section, we employed terms used in the literature; that is, we used the “HFrEF” and “HFmrEF” terms to describe our patients. The patient cohort was analysed as a whole. However, it is certainly interesting to provide details on both groups, which are now given in the Results section; please see the section “Comparison of HFrEF and HFmrEF patient subpopulations” and Supplemental Table 4.

**Reviewer #2**: Petr et al. performed a very interesting study on the prognostic value of high-sensitivity troponin I in patients with HfmrEF and HfmrEF.
The authors found s-cTnI level to be an independent marker for increased risk of an adverse prognosis.
Although limited by the exclusion of HfrEF population, the paper is well-written.
I have just several further comments to improve its quality:
1. The end point was reached only in 14% of patients (73 out of 520 patients). Perhaps it would have been compelling to have a longer follow-up and to consider hospitalizations as secondary end-point too.

Thank you for this very important comment. We used the two-year endpoint for several reasons. First, from the clinical point of view, we consider it to be a reasonable compromise between the duration of follow-up and the ability (with an appropriate probability) to predict the prognosis of heart failure patients. Second, many recent studies focusing on patients with advanced heart failure and the use of LVAD used a two-year follow-up (e.g. Ambardekar et al, J Heart Lung Transplant 2019;38:408-417; Mehra et al, N Engl J Med 2018;378:1386-1395), and we deemed the possibility of comparison with these results practical (see the section Discussion, part “Clinical benefit of the model and its interpretation”). Third, as demonstrated by the analysis of risk models in patients with hart failure, the discriminatory ability of the models for prediction of death appeared to be higher than that for prediction of death or hospitalization or prediction of hospitalization alone (p = 0.0003) (Rahimi et al, JACC Heart Fail 2014;2:440-6). Fourth – in general, the predictive accuracy of scoring models for mortality assessed by the ROC value decreases with time for which prognosis is predicted (see, for example, Littnerova et al, PloS One 2015; 10:e0123215). And fifth, although we consider the prediction of hospitalisation to be a clinically significant endpoint, both from the healthcare system point of view and from the patient’s point of view, the aim of our work was to develop a prognostic score to identify patients in which more intensive therapy, including the consideration of HTx or LVAD implantation, might change their prognosis.

If the reviewer agrees, we would like to maintain the primary endpoint as well as the duration of follow-up in the current form, as explained in the above-mentioned reasons. We also hold the opinion that adding a secondary endpoint would make the study less clearly arranged.

2. The authors generated a ROC curve of the troponin value (17 ng / L), whose patients above are at high risk. However, the population is not homogeneous (80% men), too many in NYHA 2 class (about 60%). My concerns is that this representative sample does not reflect the actual real-world HF patient population

Thank you, it is an important limitation. The demography of our cohort might be influenced by a selection bias: our group of heart failure patients has been followed up in university centres, and therefore very probably was not a representative sample of heart failure patients with EF < 50% from across the Czech Republic. A study from the Netherlands, based on an extensive registry of heart failure patients, which was intended to described the real-world population with heart failure (Brunner et all, JACC: Heart Fail 2019;7:13-21), revealed that about 77% of all patients had EF < 50%. Their mean age was 72 years (as compared to 65 years in our cohort) and men accounted for 64% only (as compared to 80,6% in our cohort).

We added this limitation to the Limitations section.

3. It might be intriguing to separate and compare patients with HfmrEF and HfrEF in two cohorts to show the difference between the two.

Thank you, we agree that this is also a very interesting point. We have therefore added it into the Results section, part “Comparison of HFrEF and HFmrEF patient subpopulations”; please see p. 14 and Supplemental Table 4.

Moreover, we removed validation of the model by the Hosmer Lemeshow test and newly the calibration of the model was assessed by a flexible calibration curve, which confirmed that the predictive model is wel calibrated (Supplemental Figure 3)
