# Supplementary material for: Prognostic value of high-sensitivity cardiac troponin I in heart failure patients with mid-range and reduced ejection fraction
Source: PLoS One. 2021 Jul 30;16(7):e0255271. doi: 10.1371/journal.pone.0255271 (PMC8323897; doi:10.1371/journal.pone.0255271)
Supplement: Attachment — Submitted filename: Response to reviewers_2.docx [file pone.0255271.s012.docx]

Reviewer #2: The paper has improved and it's almost suitable for publication.
However, I still believe that adding hospitalization as secondary end-point would be an interesting data for readers.

We agree with reviewer and we have added acute heart failure hospitalization as part of the combined secondary endpoint (death, LVAD implantation, heart transplantation and/or acute heart failure hospitalization). Including heart failure hospitalization as the component of the combined secondary endpoint leads to a diminished predictive power of increased hs-cTnI. Please, see changes in manuscript, supplemental figures (figure 1B) and supplemental tables (Supplemental Table 1, 2)
